# Ameliorative role of Atorvastatin and Pitavastatin in L-Methionine induced vascular dementia in rats

**DOI:** 10.1186/1471-2210-8-14

**Published:** 2008-08-09

**Authors:** Rajeshkumar U Koladiya, Amteshwar S Jaggi, Nirmal Singh, Bhupesh K Sharma

**Affiliations:** 1 Department of Pharmaceutical Sciences & Drug Research, Faculty of Medicine, Punjabi University, Patiala-147002, India

## Abstract

**Background:**

Statins, HMG-CoA reductase inhibitors, are widely prescribed drugs for dyslipidemias. Recent studies have indicated number of cholesterol independent actions of statins including their beneficial effects on vascular endothelial dysfunction and memory deficits associated with dementia of Alzheimer's type. However the potential of statins in dementia of vascular origin still remains to be explored. Therefore, the present study has been designed to investigate the effect of Atorvastatin & Pitavastatin on vascular endothelial dysfunction associated memory deficits in rats. In this study L-Methionine induced vascular dementia was assessed by Morris water-maze (MWM) test. Biochemical analysis was also performed to unfold possible mechanism of statins mediated modulation of vascular dementia.

**Results:**

L-Methionine produced endothelial dysfunction as reflected by significant decrease in serum nitrite concentration. L-Methionine treated rats performed poorly on MWM indicating impairment of memory as well. These rats also showed a significant rise in brain oxidative stress, acetylcholinesterase (AChE) activity and serum total cholesterol levels. Both Atorvastatin as well as Pitavastatin attenuated L-Methionine induced endothelial dysfunction associated memory deficits. Statins also reversed L-Methionine induced rise in brain oxidative stress, AChE activity and serum cholesterol.

**Conclusion:**

The beneficial effects of statins may be attributed to their multiple effects and the study highlights the potential of these drugs in vascular dementia.

## Background

Dementia of vascular origin i.e. Vascular dementia (VaD) has gained much attention in the recent times. After Alzheimer disease (AD), VaD is the second most common cause of dementia. In the vascular system, nitric oxide (NO) generated by endothelial nitric oxide synthase (eNOS) plays an important role in maintenance of vascular tone [1]. Hyperhomocysteinemia (Hhcy), or elevation of plasma total homocysteine, is an important risk factor for cardiovascular disease, stroke and vascular dementia [2-4]. Hhcy has been shown to induce endothelial dysfunction by decreasing the bioavailability of NO, and increasing vascular oxidative stress [5]. The decreased NO level has been demonstrated to contribute to the pathogenesis of dementia [6].

Increased levels of homocysteine have been documented to produce changes in structure and function of cerebral blood vessels along with oxidative stress, which play a key role in cerebral vascular dysfunction [7]. Oxidative stress and vascular dysfunction are recognized as important contributing factors in the pathogenesis of AD and other dementia of vascular origin [6]. In AD and other neurodegenerative diseases, structural deformities in the cerebral capillaries lead to impairment of cerebral perfusion with subsequent neuronal dysfunction and death [8]. The well established risk factors of endothelial dysfunction and subsequent vascular dementia such as hypertension, history of stroke, diabetes mellitus and hypercholesterolemia are all associated with high risk of AD. The noted vascular dysfunction (vascular deformities) in AD and common risk factor of AD and VaD suggest a great overlap between AD and vascular dementia [9]. Moreover, Hhcy has been documented to increase cholesterol synthesis [10]. Studies have revealed that in addition to elevated β-amyloid peptides and ApoE levels, high cholesterol level is another important risk factor for AD [11].

Only limited therapeutic interventions are available to reduce the incidence of VaD. Cholinesterase inhibitors, calcium channel blockers and glutamate antagonists are few classes of pharmacological agents which are being clinically explored to reduce symptomatically the impact of cognitive dysfunction associated with vascular dementia [12]. However, an agent that should improve both endothelial dysfunction and associated dementia still need to be explored. Very recently, the focus has been directed towards statins (HMG-CoA reductase inhibitors), which are most widely prescribed drugs for dyslipidemias [13]. Statins in additions to their cholesterol lowering action are known to possess many cholesterol independent actions including favorable effect on vascular endothelium [14]. Moreover, there is an emerging data indicating that statins exert neuroprotective and antioxidant actions [14]. Statins have been shown to reduce the risk of ischemic stroke and related memory impairment by a variety of mechanisms [15]. Epidemiological studies have suggested that individuals above 50 years of age, who were receiving statins, had a substantially lowered risk of developing dementia, independent of the presence or absence of untreated hyperlipidemia, or exposure to non-statin lipid-lowering drugs [16]. However, there are conflicting observations regarding the effect of statins on cognitive functions. Although, there are a few studies showing cognitive decline [17], some studies showing no effect on memory [18,19], yet few studies suggest improvement of cognitive functions with statin therapy. Therefore, implication of statins in endothelial dysfunction and related dementia deserves further investigation.

## Results

### Effect of Vehicle/Atorvastatin/Pitavastatin/L-Methionine on escape latency time (ELT) and time spent in target quadrant (TSTQ), using Morris water maze (MWM)

**Figure 1:**
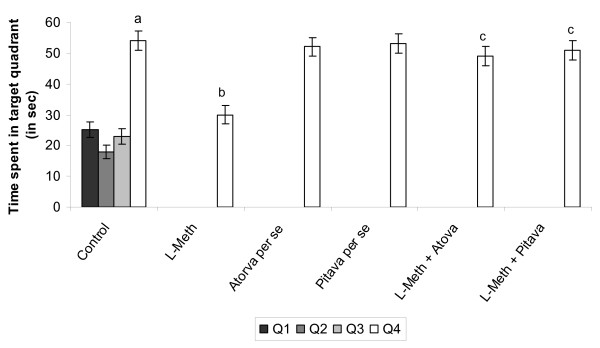
**Effect of Atorvastatin and Pitavastatin on L-Methionine induced changes in day 5 Time Spent in Target quadrant (TSTQ), using Morris Water Maze**. (L-Meth = L-Methionine; Atorva = Atorvastatin; Pitava = Pitavastatin). Each group (n = 10), represent mean ± SEM. a = *p *< 0.05 Time Spent in Q1, Q2, Q3, Quadrant Vs Q4 quadrant in Control. b = *p *< 0.05 Vs Time Spent in Target Quadrant Q4 of Control. c = *p *< 0.05 Vs Time Spent in Target Quadrant Q4 of L-Methionine treated.

Vehicle treated (0.5%w/v CMC, 10 ml/kg/p.o.) rats showed a downward trend in their ELT. There was a significant (*p *< 0.01) fall in day 4 ELT, when compared to day 1 ELT of these rats (Table 1), reflecting normal learning ability. Further on day 5 a significant (*p *< 0.01) rise in TSTQ was observed, when compared to time spent in other quadrants (Figure 1), reflecting normal retrieval as well.

**Table 1:** Effect of Atorvastatin and Pitavastatin on L-Methionine induced changes in day 4 escape latency time (ELT), using Morris Water Maze.

**Groups**	**Treatment**	**Dose (kg/day, *p.o.*)**	**ELT (day 1) in sec**	**ELT (day 4) in sec**
I	Control	10 ml(0.5%w/w CMC)	81.5 ± 4.5	20.2 ± 2.2^a^
II	L-Methionine	1.7 g	93.8 ± 4.2	49.9 ± 2.4^b^
III	Atorvastatin *per se*	10 mg	85.5 ± 4.1	22.4 ± 3.4
IV	Pitavastatin *per se*	10 mg	82.3 ± 4.3	21.3 ± 3.8
V	L-Methionine + Atorvastatin	10 mg	81.9 ± 3.9	27.0 ± 2.4^c^
VI	L-Methionine + Pitavastatin	10 mg	82.5 ± 4.4	27.9 ± 3.6^c^

Each group (n = 10), represent mean ± SEM.

a = *p *< 0.01 Day 1 Vs Day 4 ELT in vehicle control.

b = *p *< 0.05 Vs Day 4 ELT in vehicle control.

c = *p *< 0.05 Vs Day 4 ELT in L-Methionine treated group.

Administration of Atorvastatin (10 mg/kg/p.o., 2 weeks and 4 day)/Pitavastatin (10 mg/kg/p.o., 2 weeks and 4 day) did not show any significant per se effect on ELT and TSTQ as compared to vehicle treated rats. (Table 1 and Figure 1) L-Methionine (1.7 g/kg/p.o., 4 weeks and 4 day) administration produced a significant increase (*p *< 0.05) in day 4 ELT, when compared to day 4 ELT of vehicle control (Table 1) indicating impairment of acquisition. Further L-Methionine administration also produced a significant (*p *< 0.01) decrease in TSTQ, when compared TSTQ of vehicle control animals (Figure 1), indicating impairment of memory as well.

### Effect of Atorvastatin/Pitavastatin on L-Methionine induced impairment of learning and memory using Morris water maze

Administration of Atorvastatin (10 mg/kg/p.o., 2 weeks and 4 day), Pitavastatin (10 mg/kg/p.o., 2 weeks and 4 day), significantly (*p *< 0.05) prevented L-Methionine induced rise in day 4 ELT, indicating reversal of L-methionine induced impairment of acquisition (Table 1). Further treatment of these drugs also attenuated L-Methionine induced decrease in day 5 TSTQ in a significant manner (*p *< 0.05), indicating reversal of L-methionine induced impairment of memory (Figure 1).

### Effect of Atorvastatin/Pitavastatin on L-Methionine induced change in endothelium dependent relaxation

**Figure 2:**
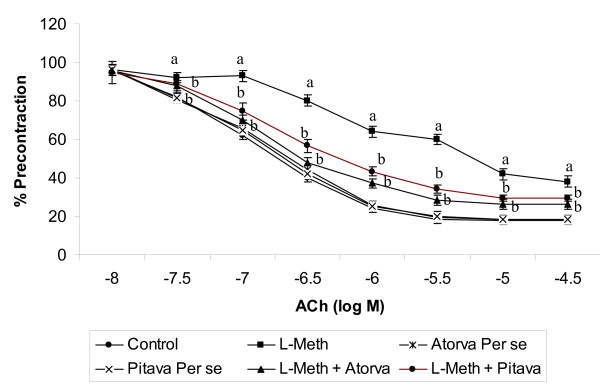
**Effect of Atorvastatin/Pitavastatin on L-Methionine change in endothelium dependent relaxation using isolated rat aortic ring preparation**. (L-Meth = L-Methionine; Atorva = Atorvastatin; Pitava = Pitavastatin). Each group (n = 10), represent mean ± SEM. Responses are expressed as % of maximum contraction induced by phenylephrine (3 × 10^-6 ^M). a = *p *< 0.05 Vs Control. b = *p *< 0.05 Vs L-Methionine treated group.

**Figure 3:**
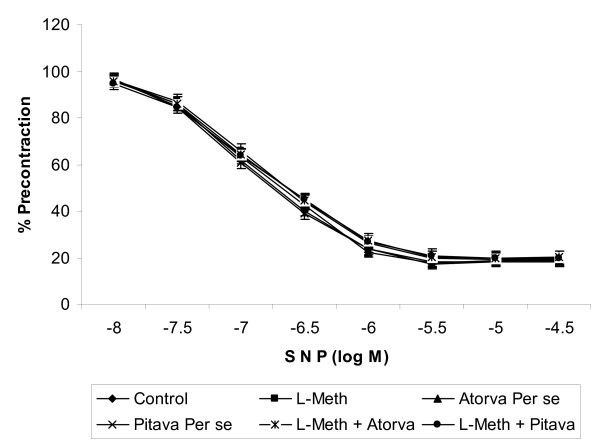
**Effect of Atorvastatin/Pitavastatin/L-Methionine induced on sodium nitroprusside induced endothelium independent relaxation using isolated rat aortic ring preparation**. (L-Meth = L-Methionine; Atorva = Atorvastatin; Pitava = Pitavastatin). Each group (n = 10), represent mean ± SEM. Responses are expressed as % of maximum contraction induced by phenylephrine (3 × 10^-6 ^M).

Acetylcholine (ACh) and sodium nitroprusside (SNP) in a dose dependent manner produced endothelium dependent and independent relaxation in phenylephrine (3 × 10^-6 ^M) precontracted isolated rat aortic ring preparation. L-methionine (1.7 g/kg/*p.o*., 4 weeks and 4 day) administration significantly (*p *< 0.05) attenuated acetylcholine induced endothelium dependent relaxation (Figure 2), however it did not affect SNP induced endothelium independent relaxation (Figure 3). Treatment of Atorvastatin (10 mg/kg/p.o., 2 weeks and 4 day), Pitavastatin (10 mg/kg/p.o., 2 weeks and 4 day), significantly (*p *< 0.05) abolished the effect of L-Methionine on endothelial dependent relaxation. Further Atorvastatin and Pitavastatin did not show any per se effect on endothelium dependent relaxation.

### Effect of Atorvastatin/Pitavastatin on L-Methionine induced change in serum homocysteine level

Administration of L-Methionine (1.7 g/kg/p.o., 4 weeks and 4 day), produced a significant (*p *< 0.01) increase in serum homocysteine, when compared to vehicle treated rats. Treatment with Atorvastatin (10 mg/kg/p.o., 2 weeks and 4 day)/Pitavastatin (10 mg/kg/p.o., 2 weeks and 4 day) produced a significant (*p *< 0.05) reduction of L-methionine induced rise in serum homocysteine level (Table 2). Further, Atorvastatin (10 mg/kg/p.o., 2 weeks and 4 day)/Pitavastatin (10 mg/kg/p.o., 2 weeks and 4 day) did not show any significant *per se *effect on serum homocysteine level (Table 2).

**Table 2:** Effect of Atorvastatin and Pitavastatin on L-Methionine induced changes in Serum homocysteine.

**Groups**	**Treatment**	**Dose (kg/day, *p.o.*)**	**Serum homocysteine (μM)**
I	Control	10 ml(0.5%w/w CMC)	4.23 ± 0.15
II	L-Methionine	1.7 g	20.8 ± 0.84^a^
III	Atorvastatin *per se*	10 mg	3.95 ± 0.21
IV	Pitavastatin *per se*	10 mg	3.92 ± 0.23
V	L-Methionine + Atorvastatin	10 mg	12.9 ± 0.38^b^
VI	L-Methionine + Pitavastatin	10 mg	13.5 ± 0.34^b^

Each group (n = 10), represent mean ± SEM.

a = *p *< 0.01 Vs serum homocysteine level of Control.

b = *p *< 0.05 Vs serum homocysteine level of L-Methionine treated group.

### Effect of Atorvastatin/Pitavastatin on L-Methionine induced change in serum nitrite level

**Figure 4:**
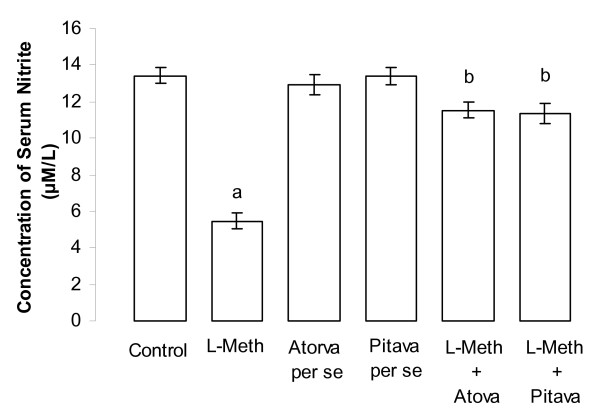
**Effect of Atorvastatin and Pitavastatin on L-Methionine induced changes in serum nitrite level**. (L-Meth = L-Methionine; Atorva = Atorvastatin; Pitava = Pitavastatin). Each group (n = 10), represent mean ± SEM. a = *p *< 0.01 Vs serum nitrite of Control. b = *p *< 0.05 Vs serum nitrite of L-Methionine treated group.

Administration of L-Methionine (1.7 g/kg/p.o., 4 weeks and 4 day), produced a significant (*p *< 0.01) decrease in serum nitrite, when compared to vehicle treated rats. Treatment with Atorvastatin (10 mg/kg/p.o., 2 weeks and 4 day)/Pitavastatin (10 mg/kg/p.o., 2 weeks and 4 day) prevented L-methionine induced decrease in serum nitrite level in a significant (*p *< 0.05) manner (Figure 4). Further, Atorvastatin (10 mg/kg/p.o., 2 weeks and 4 day)/Pitavastatin (10 mg/kg/p.o., 2 weeks and 4 day) did not show any significant *per se *effect on serum nitrite level (Figure 4).

### Effect of Atorvastatin/Pitavastatin/L-Methionine induced change in total serum cholesterol levels

**Figure 5:**
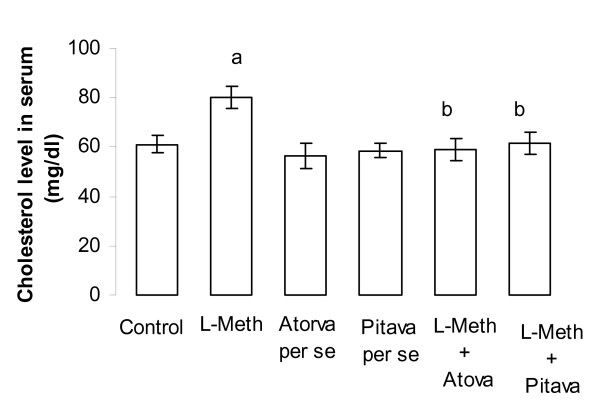
**Effect of Atorvastatin and Pitavastatin on L-Methionine induced changes in serum total cholesterol level**. (L-Meth = L-Methionine; Atorva = Atorvastatin; Pitava = Pitavastatin). Each group (n = 10), represent mean ± SEM. a = *p *< 0.05 Vs serum total cholesterol of Control. b = *p *< 0.05 Vs serum total cholesterol of L-Methionine treated.

Administration of L-Methionine (1.7 g/kg/p.o., 4 weeks and 4 day) produced a significant (*p *< 0.05), increase in total serum cholesterol levels of animals, when compared to vehicle control. Treatment with Atorvastatin (10 mg/kg/p.o., 2 weeks and 4 day)/Pitavastatin (10 mg/kg/p.o., 2 weeks and 4 days) attenuated L-Methionine induced rise in total serum cholesterol levels in a significant (*p *< 0.05) manner (Figure 5). Furthermore Atorvastatin (10 mg/kg/p.o., 2 weeks and 4 day)/Pitavastatin (10 mg/kg/p.o., 2 weeks and 4 day) did not show any significant *per se *effect on total serum cholesterol levels (Figure 3), when compared to vehicle control group (Figure 5).

### Effect of Atorvastatin/Pitavastatin on L-Methionine induced change in brain acetyl cholinesterase (AChE) activity

**Figure 6:**
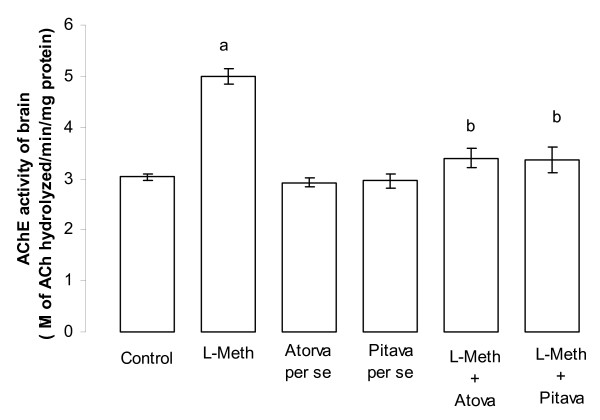
**Effect of Atorvastatin and Pitavastatin on L-Methionine induced changes brain acetylcholinesterase (AChE) activity**. (L-Meth = L-Methionine; Atorva = Atorvastatin; Pitava = Pitavastatin). Each group (n = 10), represent mean ± SEM. a = *p *< 0.05 brain AChE activity of Control. b = *p *< 0.05 Vs brain AChE activity of L-Methionine treated.

Administration of L-Methionine (1.7 g/kg/p.o., 4 weeks and 4 day) produced a significant (*p *< 0.05), increase in brain AChE activity, when compared to vehicle treated rats. Treatment with Atorvastatin (10 mg/kg/p.o., 2 weeks and 4 day)/Pitavastatin (10 mg/kg/p.o., 2 weeks and 4 day) significantly (*p *< 0.05), prevented L-Methionine induced rise in brain AChE activity. Further Atorvastatin (10 mg/kg/p.o., 2 weeks and 4 day)/Pitavastatin (10 mg/kg/p.o., 2 weeks and 4 day) did not show any significant *per se *effect on brain AChE activity (Figure 6).

### Effect of Atorvastatin/Pitavastatin on L-Methionine induced change in oxidative stress levels of brain

**Figure 7:**
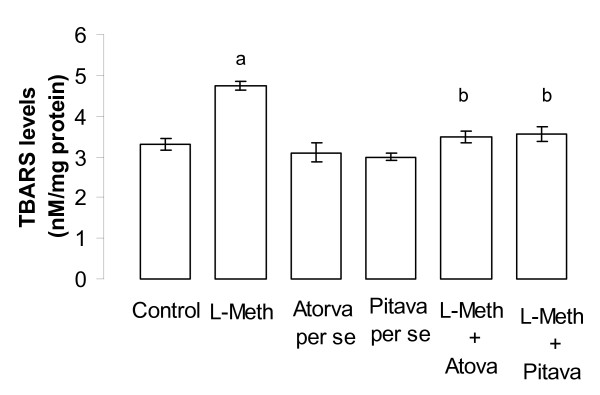
**Effect of Atorvastatin and Pitavastatin on L-Methionine induced changes in brain thiobarbituric acid reactive species (TBARS) level**. (L-Meth = L-Methionine; Atorva = Atorvastatin; Pitava = Pitavastatin). Each group (n = 10), represent mean ± SEM. a = *p *< 0.01 brain TBARS level of Control. b = *p *< 0.05 Vs brain TBARS level of L-Methionine treated group.

**Figure 8:**
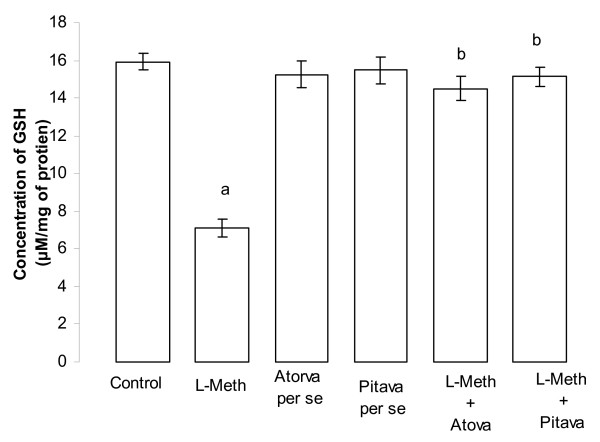
**Effect of Atorvastatin and Pitavastatin on L-Methionine induced changes in brain reduced glutathione (GSH) level**. (L-Meth = L-Methionine; Atorva = Atorvastatin; Pitava = Pitavastatin). Each group (n = 10), represent Mean ± SEM. a = *p *< 0.01 brain GSH level of Control. b = *p *< 0.05 Vs brain GSH level of L-Methionine treated group.

Administration of L-Methionine (1.7 g/kg/p.o., 4 weeks and 4 day), produced a significant increase (*p *< 0.05), in brain thiobarbituric acid reactive species (TBARS) level (Figure 7) and a decrease (*p *< 0.01), in the level of reduced form of glutathione (GSH) (Figure 8), when compared to vehicle treated rats; hence reflecting induction of oxidative stress. Treatment with Atorvastatin (10 mg/kg/p.o., 2 weeks and 4 day)/Pitavastatin (10 mg/kg/p.o., 2 weeks and 4 day) significantly (*p *< 0.05) prevented L-Methionine induced oxidative stress (Figure 7, 8). Further Atorvastatin (10 mg/kg/p.o., 2 weeks and 4 day)/Pitavastatin (10 mg/kg/p.o., 2 weeks and 4 day) did not show any significant *per se *effect on oxidative stress level (Figure 7, 8).

## Discussion

Young male rats were employed in the present study, as it is reported that aging and consequent variation of estrogen in blood modulates the activity of endothelial nitric oxide synthase (eNOS), which further affects the function of vascular endothelium and memory [20,21]. Morris Water Maze test employed in present study is one of the most widely accepted models to evaluate learning and memory of the animals [22,23]. Nitric oxide (NO) synthesized in the endothelium and its levels get attenuated during endothelial dysfunction. Endogenously formed NO is highly unstable and gets converted to nitrate and nitrite [24]. Therefore, serum nitrite concentration has been employed as specific a marker of endothelial dysfunction [5].

A significant decrease in escape latency time (ELT day 4) of control animals during ongoing acquisition trials denoted normal acquisition of memory and an increase in time spent in target quadrant (TSTQ), in search of missing platform during retrieval trial indicated, retrieval of memory. These results are consistent to our earlier findings and reports from other laboratory [13,25,26].

L-Methionine treatment for 4 weeks, in the present study significantly raised serum homocysteine level, decreased serum nitrite levels and markedly attenuated acetylcholine induced endothelium dependent relaxation, therefore, reflecting endothelial dysfunction. Further L-methionine administration also produced a significant impairment of acquisition and retrieval of memory as reflected by decreased Morris water-maze performance. Moreover, an enhancement of brain acetyl cholinesterase (AChE) activity, increase in brain oxidative stress (as reflected by rise in brain TBARS and fall in GSH levels) and increase in serum total cholesterol levels were also observed. Recently, it has been reported that chronic experimental hyperhomocysteinemia produce cognitive dysfunction [27,28], and increase in brain AChE activity [29]. L-Methionine induced hyperhomocysteinemia is a well established model of experimental endothelial dysfunction [30,31]. Hyperhomocysteinemia has been reported to induce endothelial dysfunction by decreasing the bioavailability of NO and by increasing vascular oxidative stress [5]. Our observation also supports above contention, as a significant fall in serum nitrite levels along with rise in oxidative stress levels (increase TBARS and decrease GSH) were noted in L-methionine treated rats.

The increased level of homocysteine has been reported to produce change in structure and function of cerebral blood vessels along with oxidative stress, which play a key role in cerebral vascular dysfunction [7]. Several lines of evidences have strongly advocated a direct relationship between vascular endothelial dysfunction and dementia better known as vascular dementia [32,33]. Cerebral vascular endothelial dysfunction has also been shown to enhance progression of dementia of Alzheimer disease (AD) [33]. Enhanced levels of brain AChE activity and oxidative stress have also been noted in patients suffering form dementia of AD and other dementias [34].

Further hyperhomocysteinemia has also been shown to be neurotoxic, and the neurotoxicity may be due to over activation of N-methyl-D-aspartate receptors or by enhanced vulnerability of hippocampal neuron to excitotoxic insults and amyloid β-peptide toxicity [35,36]. Moreover, methionine rich diet in rats has been demonstrated to enhance cholesterol concentration in the plasma and liver [10]. Several studies have also revealed high serum cholesterol level as another important risk factor of AD [37]. Therefore L-Methionine induced memory dysfunction in the present study may be attributed to its multiple effects i.e. decrease in serum nitrite level (endothelial dysfunction), rise in oxidative stress level, enhancement of brain AChE activity, serum total cholesterol as well as direct neurotoxicity.

In the present study, treatment with Atorvastatin and Pitavastatin significantly improved L-Methionine induced endothelial dysfunction manifested in the terms of endothelium dependent relaxation; increased serum nitrite levels, decreased serum homocysteine, and decreased oxidative stress (decrease TBARS and increase GSH). Statins, in addition to their cholesterol lowering action has been reported to exert number of cholesterol independent actions i.e. pleotropic actions. Statins have been demonstrated to enhance the expression of eNOS in human endothelial cells [38]. They have been known to activate Akt/protein kinase B, which subsequently activates eNOS [39]. Studies with Atorvastatin and Simvastatin have shown to inhibit the expression of prepro-endothelin (ET)-1 mRNA and reduce plasma ET-1 levels, endothelin being a potent vasoconstricting agent [40]. Furthermore, statins appear to inhibit the synthesis of isoprenoids, compounds that are required for the posttranslational modification of important signaling molecules such as Rho, Rac, and Ras [41]. Inhibition of Rho activation has been shown to increase endothelial NO production [38] and reduces ET-1 expression [40]. Recently, Atorvastatin has been documented to improve the function of endothelium by lowering the expression of p22phox and production of reactive oxygen species [30,42]. Further, in another recent report it has been indicated that hyperhomocysteinemia induce impairment of NO production through the modulation of Cav-1 expression associated with a loss of eNOS in caveolae [43]. Statins have been shown to prevent the expression of caveolin, a negative regulator of eNOS [44]. Therefore Atorvastatin and Pitavastatin induced improvement of endothelial dysfunction in our study may be attributed to their stimulatory effect on endothelial nitric oxide production and their antioxidative action.

Further, administration of Atorvastatin/Pitavastatin in the present investigation also, reversed memory deficit induced by L-Methionine (hyperhomocysteinemia). Several recent clinical reports have suggested that the net brain cholesterol concentration is regulated by serum cholesterol level and there is a cross talk between the CNS and peripheral cholesterol pools [45,46]. Cholesterol turnover appears to play a crucial role in the deposition and clearance of amyloid peptide in brain and ApoE is a cholesterol transporting protein that is associated with amyloid deposits [47,48]. Further, serum cholesterol, atherosclerosis, apolipoprotein-E and AD all appear to be interconnected [49,50]. Studies involving cultured rat cortical neurons have revealed neuroprotective action of Atorvastatin against glutamate induced excitotoxicity [51].

In our recent studies, we have reported that Atorvastatin, Simvastatin and Pitavastatin reversed memory deficits of rats and mice associated with dementia of AD type and memory improving effects of these statins mediated through their cholesterol dependent and in dependent actions [13,25]. Furthermore, statins have also been shown to exert beneficial effects in cerebral ischemia and stroke providing neuroprotection via enhancement of NO production [52].

Many studies, in the recent years have implicated a vital role for NO in neurophysiological process of learning and memory [53]. Inhibition of NO system impaired memory in rats [54,55]. Whereas, stimulation of NO production improved cognitive functions in Alzheimer patients [56]. NO, donors like molsidomine reversed scopolamine induced amnesia in rats [57]. NO probably, acts as retrograde messenger in the formation of long term potentiation (LTP) at the molecular level of learning and memory processes [58]. Therefore, Atorvastatin and Pitavastatin in present investigation appear to reverse L-Methionine induced memory deficits via multiple actions viz; decrease in total cholesterol, brain oxidative stress (decrease TBARS and increase GSH) levels, AChE activity and increase in serum nitrite levels. Nevertheless, further studies incorporating female rats as well as other species of animals such as mice, using different memory model (other than Morris water-maze), duly supported by histopathology of brain tissue are required in order to support and extend potential of these statins in endothelial dysfunction related memory deficits.

## Conclusion

It may be concluded that chronic L-Methionine administration (hyperhomocysteinemia) has produced endothelial dysfunction along with impairment of learning and memory (vascular dementia). Atorvastatin and Pitavastatin exerted beneficial effects on endothelial dysfunction and related memory deficits by virtue of their cholesterol dependent as well as cholesterol independent actions. Perhaps this is the first report highlighting potential of Statins in L-Methionine induced endothelial dysfunction associated memory deficits.

## Methods

### Animals

Age matched (six months old) male Wistar Albino rats, weighing 150–200 g were employed in the present study. Animals were procured from Institute of Veterinary Science, Izat-Nagar Bareilly (U.P), India. Rats were provided standard laboratory feed (Kisan Feeds Ltd, Chandigarh, India) and tap water ad libitum and were exposed to 12 h light and 12 h dark cycle. The animals were acclimatized to the laboratory condition before experiments. The experimental protocol was duly approved by Institutional Animal Ethics Committee (IAEC) and care of the animals was taken as per guidelines of the Committee for the Purpose of Control and Supervision of Experiments on Animals (CPCSEA), ministry of Environment and Forests, Government of India, (Reg. No. 107/1999/CPCSEA).

### Drugs and Chemicals

Atorvastatin was a gift from Ind swift Ltd., Mohali, Punjab, India. Pitavastatin was a gift from Zydus Research Center, Ahmedabad, Gujarat, India. All other reagents purchased from Merck limited, Mumbai, India, SD fine-chemicals limited, Mumbai, India, Loba Chem, Mumbai, India and Sigma-Aldrich, USA. Atorvastatin, Pitavastatin and L-Methionine were suspended in 0.5% w/v of carboxy methyl cellulose (CMC).

### L-Methionine Induced endothelial dysfunction and Vascular Dementia

Rats, were administered L-Methionine (1.7 g/kg/day, p.o.) for 4 weeks to produce hyperhomocysteinemia-induced endothelial dysfunction [30]. Assessment of vascular endothelial function was carried out by measuring acetylcholine induced endothelium dependent relaxation and sodium nitroprusside induced endothelium independent relaxation using isolated aortic ring preparation according to the method of Pieper [59] together with estimation of serum nitrite concentration.

Body weight of rats was monitored weekly. After 4 weeks, rats were subjected to Morris water maze test for the evaluation of their memory status. The L-Methionine treatment was continued during acquisition trials on Morris Water Maze.

### Morris Water Maze Test

Morris Water Maze (MWM) test was employed to assess learning and memory of rats [22,23]. The MWM procedure was based on a principle, where the animals were placed in a large pool of water, as animals dislike swimming, their tendency was to escape from the water being accomplished by finding an escape platform. MWM consisted of large circular pool (150 cm in diameter, 45 cm in height), filled to a depth of 30 cm with water at 28 ± 1°C. The water was made opaque with non-toxic white colored dye. The tank was divided hypothetically, into four equal quadrants with help of two threads, fixed at right angle to each other on the rim of the pool. A submerged platform (10 cm^2^), painted in white was placed inside the target quadrants of this pool, 1 cm below surface of water. The position of platform was kept unaltered throughout the training session. Each animal was subjected to four consecutive trials on each day with gap of 5 min. The rat was gently placed in the water of the pool between quadrants, facing the wall of pool with drop location changing for each trial, and allowed 120 sec to locate submerged platform. Then, it was allowed to stay on the platform for another 20 sec. If it failed to find the platform within 120 sec, it was guided gently onto platform and allowed to remain there for 20 sec. Escape latency time (ELT) to locate the hidden platform in water maze was noted as an index of acquisition or learning. Animal was subjected to four acquisition trials daily for four consecutive days. On fifth day, the platform was removed and each rat was allowed to explore the pool for 120 sec. Mean time spent in all four quadrants was noted. The mean time spent by the animal in target quadrant searching for the hidden platform was noted as an index of retrieval.

#### Acquisition Trial

Each rat was subjected to four trials on each day. A rest period of 5 min was allowed in between each trial. Four trials per day were repeated for four consecutive days. Starting position on each day to conduct four acquisition trials was changed as described below and Q4 was maintained as target quadrant in all acquisition trials. Mean escape latency time (ELT) calculated for each day during acquisition trials and day 4 ELT was used as an index of acquisition (table 3).

**Table 3:** Mean escape latency time (ELT) calculated for each day during acquisition trials and day 4 ELT was used as an index of acquisition

Day1		Q1		Q2		Q3		Q4
Day2		Q2		Q3		Q4		Q1
Day3		Q3		Q4		Q1		Q2
Day4		Q4		Q1		Q2		Q3

#### Retrieval Trial

On fifth day the platform was removed. Rat was placed in water maze and allowed to explore the maze for 120 sec. Each rat was subjected to four such trials and each trial was started from different quadrant. Mean time spent in all three quadrants i.e. Q1, Q2 and Q3 were recorded and the time spent in the target quadrant i.e. Q4 in search of missing platform provided an index of retrieval. The experimenter always stood at the same position. Care was taken not to disturb the relative location of water maze with respect to other objects in the laboratory serving, as prominent visual clues. All the trials were completed between 09.00 to 17.00 hrs in semi sound proof laboratory.

### Biochemical Parameters

#### Collection of sample

The animals were sacrificed by cervical dislocation; thoracic aorta and brain tissue were carefully removed. Thoracic aorta was used for endothelium dependent and independent relaxation, whereas brain tissue was subjected to various biochemical estimations.

The removed brains were homogenized in phosphate buffer (pH 7.4, 10% w/v) using Teflon homogenizer. The clear supernatant, obtained after centrifugation at 3000 rpm for 15 min, was used to estimate acetyl cholinesterase (AChE) activity, thiobarbituric acid reactive species (TBARS), reduced glutathione (GSH) and protein content.

Blood samples for biochemical estimation were collected just before sacrificing the rats. The blood was kept at room temperature for 30 min and then centrifuged at 4000 rpm for 15 min to separate serum. Serum was used to estimate serum homocysteine, serum nitrite concentration and total serum cholesterol.

#### Estimation of serum homocysteine

Determination of homocysteine was carried out using HPLC (Varian Inc., CA, USA) attached with fluorescent HPLC detector according to the method of *Dimitrova et al *[60]. 100 μl of serum sample was added to 1.5 ml eppendorff containing 10 μl of water and 5 μl of n-amyl alcohol and was gently vortexed. 35 μl of sodium borohydrate reagent (35 μl of 1.43 M sodium borohydride in 0.1 M sodium hydroxide), 35 μl of 1 M of hydrochloric acid and 50 μl of 10 mM monobromobimane in 4 M sodium EDTA (pH 7) were added to eppendorff tube containing serum sample, capped, mixed and incubated at 42°C for 12 min. Then, it was cooled, vortexed and maintained at RT for 10 min. The sample was centrifuged at 12,200 g for 10 min to remove protein, acidic clear supernatant was separated and supernatant was adjusted to pH 4 using 25 μl of 2 M Tris-HCL. The sample was then centrifuged at 12,200 g for 1 min and 100 μl of supernatant was aliquoted for HPLC analysis.

A fixed volume autosampler was used to inject 20 μl of sample into 4.6 × 250 mm RP8 ultrasphere column equipped with brownlee RP 18 new guard column. The solvent mixture in ratio of 94.75: 5: 0.25 of water: methanol: acetic acid was maintained at pH 3.4 using 5 M NaOH in pump A and 100% methanol was maintained in pump B. The injection rate of sample was maintained at 2 ml per min. HPLC detector was set with excitation wavelength at 390 nm and emission wavelength at 418 nm. The sensitivity range and rise time of detector were set at 0.1 sec and 2 sec respectively. The calibration curve for homocysteine as homocysteine-S-bimane was plotted using 100 μl quality control serum fortified with 10 μl homocysteine (1–1000 μM) solution. The data of calibration curve were regressed and the curve was used to calculate serum concentration of homocysteine.

Rats with serum homocysteine levels of > 10 μM were considered to be hyperhomocysteinemic.

#### Estimation of serum nitrite concentration

Serum nitrite concentration was estimated using method of Sastry *et al. *[24]. 400 μl of carbonate buffer (pH 9.0) was added to 100 μl of serum or standards sample followed by addition of small amount (~0.15 g) of copper-cadmium alloy. The tubes were incubated at room temperature for 1 h to reduce nitrate to nitrite. The reaction was stopped by adding 100 μl of 0.35 M sodium hydroxide. Following this, 400 μl of zinc sulfate solution (120 mM) was added to deproteinate the serum samples. The samples were allowed to stand for 10 min and then centrifuged at 4000 g for 10 min. Greiss reagent (250 μl of 1.0% sulfanilamide prepared in 3 N HCl and 250 μl of 0.1% N-naphthylethylenediamine prepared in water) was added to aliquots (500 μl) of clear supernatant and serum nitrite was measured spectrophotometrically (DU 640B Spectrophotometer, Beckman Coulter Inc., CA, USA) at 545 nm. The standard curve of sodium nitrite (5 to 50 μM) was plotted to calculate concentration of serum nitrite.

#### Estimation of total cholesterol

Total serum cholesterol was estimated spectrophotometrically (DU 640B spectrophotometer, Beckman Coulter Inc., CA, USA) at 540 nm by CHOP/POD-phosphotungstate enzymatic method [61] using commercially available conventional diagnostic kit (Monozyme India ltd., Secundrabad).

#### Estimation of brain acetyl cholinesterase (AChE) activity

The whole brain AChE activity was measured by the method of Ellman *et al *[62] with slight modifications [63]. This was measured on the basis of the formation of yellow colour due to the reaction of thiocholine with dithiobisnitrobenzoate ions. The rate of formation of thiocholine from acetylcholine iodide in the presence of brain cholinesterase was measured using a spectrophotometer. 0.5 ml of supernatant liquid of the brain homogenate was pipetted out into 25 ml volumetric flask and dilution was made with a freshly prepared DTNB {5,5'-dithiobis (2-nitro benzoic acid)} solution (10 mg DTNB in 100 ml of sorenson phosphate buffer, pH 8.0). From the volumetric flask, two 4 ml portions were pipetted out into two test tubes. Into one of the test tube, 2 drops of eserine solution was added. 1 ml of substrate solution (75 mg of acetylcholine iodide per 50 ml of distilled water) was pipetted out into both of the test tubes and incubated for 10 min. at 30°C. The solution containing eserine solution was used for zeroing the colorimeter and change in absorbance per min. of the sample was read spectrophotometrically (DU 640B spectrophotometer, Beckman Coulter Inc., CA, USA) at 420 nm. AChE activity was calculated using the following formula:

$$\text{R}=\frac{\delta \text{ O}.\text{D}.\times \text{Volume of Assay }(3\text{ ml})}{\text{E}\times \text{mg of protein}}$$

Where R = rate of enzyme activity in 'n' mole of acetylcholine iodide hydrolyzed/minute/mg protein

δO.D. = Change in absorbance/minute

E = Extinction coefficient = 13600/M/cm

#### Estimation of brain thiobarbituric acid reactive species (TBARS) level

The whole brain TBARS level was measured by the method of Ohokawa *et al *[64] with slight modifications. 0.2 ml brain homogenate was pipetted out in a test tube, followed by addition of 0.2 ml sodium dodecyl sulphate (SDS), 1.5 ml of 30% acetic acid (pH-3.5), 1.5 ml of 0.8% thiobarbituric acid (TBA) and make up the volume up to 4.0 ml with distilled water (DW). The test tubes were incubated at 95°C for 60 min., and then cool it. 1.0 ml of DW and 5.0 ml of n-butanol: pyridine (15: 1 v/v) mixture was added to the test tubes and centrifuged at the 4,000 × g for 10 min. The absorbance of developed colour in organic layer was Measured spectrophotometrically at 532 nm (DU 640B spectrophotometer, Beckman Coulter Inc., CA, USA). The absorbance from a standard curve generated using 1,1,3,3, tetra-methoxy propane as standard (range = 1 nmol – 10 nmol) was extrapolated.

#### Estimation of brain reduced glutathione (GSH) level

The whole brain GSH level was measured by the method of Beutler *et al *[65] with slight modifications. Tissue homogenate was taken and the proteins were precipitated with 10% w/v chilled trichloroacetic acid. Samples were kept in ice bath and were centrifuged after 30 min. at 1000 × g for 10 min. at 4°C. GSH levels were measured in the supernatant. 0.5 ml supernatant was mixed with 2.0 ml of 0.3 M disodium hydrogen phosphate solution and 0.25 ml of freshly prepared DTNB {5,5'-dithiobis (2-nitro benzoic acid)} solution (40 mg/100 ml in 1% w/v sodium citrate) was added just before measuring the absorbance spectrophotometrically at 412 nm (DU 640B spectrophotometer, Beckman Coulter Inc., CA, USA). Different concentration of GSH standard was also processed similarly to prepare a standard curve (5–50 μg) simultaneously. Results have been expressed as n mole of GSH/mg of protein.

#### Estimation of brain total protein

For the estimation of total protein in brain, method of Lowry *et al *[66] with slight modifications was used. 150 μL of supernatant was taken in a test tube, volume was made up to 1 ml with distilled water than 5 ml of Lowry's reagent (freshly prepared mixture of 1% w/v copper sulphate, 2% w/v sodium potassium tartrate and 2% w/v sodium carbonate in 0.1 N NaOH in the ratio of 1:1:98 respectively), was added and mixed thoroughly. Mixture was allowed to stand for 15 minutes at room temperature and then 0.5 ml of 1:1 v/v diluted Folin-Ciacalteu reagent was added. Contents were vortexed and incubated at 37°C for 30 minutes. Then absorbance was determined spectrophotometrically at 750 nm (DU 640B spectrophotometer, Beckman Coulter Inc., CA, USA) against suitably prepared blank. A standard curved using 25–200 mg of BSA was plotted. The amount of total protein was expressed in mg.

### Experimental Design

Six groups, each group comprised 10 albino Wistar rats, were employed in the present study.

#### Group I (Vehicle treated control)

Rats were administered 0.5% w/v CMC (10 ml/kg/day, p.o.) for 4 weeks and then subjected to MWM test. The vehicle was also administered 45 min before acquisition trial conducted from day 1 to day 4 and retrieval trial conducted on day 5.

#### Group II (L-Methionine treated)

In order to induce hyperhomocysteinemia, the rats were administered L-methionine (1.7 g/kg/day, p.o.) for 4 weeks and then subjected to MWM test. The treatment of L-Methionine was continued (administered 45 min before) during acquisition trial conducted from day 1 to day 4. The animals were administered vehicle (0.5%w/v CMC, 10 ml/kg, p.o.) 45 min before retrieval trial conducted on day 5.

#### Group III (Atorvastatin *per se*)

Rats were administered Atorvastatin (10 mg/kg/day, p.o.) for 2 weeks and then subjected to MWM test. The treatment was continued (administered 45 min before) during acquisition trial conducted from day 1 to day 4. The animals were administered vehicle (0.5%w/v CMC, 10 ml/kg, p.o) 45 min before retrieval trial conducted on day 5.

#### Group IV (Pitavastatin *per se*)

Rats were administered Pitavastatin (10 mg/kg/day, p.o.) for 2 weeks and rest of protocol was same as mentioned in group III.

#### Group V (L-Methionine + Atorvastatin treated)

The hyperhomocysteinemic rats were treated with Atorvastatin (10 mg/kg/day, p.o.) for 2 weeks (3^rd ^and 4^th ^week of L-Methionine administration) and then subjected to MWM test. The co-administration of Atorvastatin and L-Methionine was continued (administered 45 min before) during acquisition trial conduct from day 1 to day 4. The animals were administered vehicle (0.5%w/v CMC, 10 ml/kg, p.o) 45 min before retrieval trial conducted on day 5.

#### Group VI (L-Methionine + Pitavastatin treated)

The hyperhomocysteinemic rats were treated with Pitavastatin (10 mg/kg/day, p.o.) and subjected to MWM test as described in group VI.

### Statistical analysis

The results were expressed as mean ± standard error of means (S.E.M.) The data for isolated aortic ring preparation was statistically analyzed using repeated measure ANOVA followed by Newman-Keul's test. Rest of the data obtained from various groups was statistically analyzed using one-way ANOVA followed by Tukey's Multiple Range test. The *p *< 0.05 was considered to be statistically significant.

## Authors' contributions

RUK carried out surgical operations, behavioral tests, and biochemical tests the data analysis in animal studies. BKS assisted in carrying out surgery, behavioral tests, and biochemical tests. ASJ carried out data analysis and participated in critical intellectual discussion and designing of the experiments. NS conceived the idea, coordinated the study, carried our data interpretation and drafted the manuscript. All authors read and approved the final manuscript.
